# Leukemic Involvement Is a Common Feature in Waldenström Macroglobulinemia at Diagnosis

**DOI:** 10.3390/cancers15164152

**Published:** 2023-08-17

**Authors:** Sara Montesdeoca, Nieves García-Gisbert, Xavier Calvo, Leonor Arenillas, David Román, Concepción Fernández-Rodríguez, Rosa Navarro, Beatriz Costan, María del Carmen Vela, Laura Camacho, Eugènia Abella, Lluís Colomo, Marta Salido, Anna Puiggros, Lourdes Florensa, Blanca Espinet, Beatriz Bellosillo, Ana Ferrer del Álamo

**Affiliations:** 1 Laboratori d’Hematologia, Servei Diagnòstic de Laboratori, Hospital Sant Joan de Déu, Esplugues de Llobregat, 08950 Barcelona, Spain; sara.montesdeoca@sjd.es; 2 Grup de Recerca Translacional en Neoplasies Hematològiques (GRETNHE), Hospital del Mar Research Institute (IMIM), 08003 Barcelona, Spain; xcalvo@psmar.cat (X.C.); larenillas@psmar.cat (L.A.); oroman@psmar.cat (D.R.); rmnavarroa@psmar.cat (R.N.); bcostan@psmar.cat (B.C.); lcolomo@psmar.cat (L.C.); msalido@psmar.cat (M.S.); apuiggros@psmar.cat (A.P.); lflorensa@psmar.cat (L.F.); bespinet@psmar.cat (B.E.); 3 Laboratori de Biologia Molecular, Servei de Patologia, Hospital del Mar, 08003 Barcelona, Spain; nieves.garciagisbert@gmail.com (N.G.-G.); mconcepcionfernandezrodriguez@psmar.cat (C.F.-R.); mvela@psmar.cat (M.d.C.V.); lcamacho@psmar.cat (L.C.); bbellosillo@psmar.cat (B.B.); 4 Grup de Recerca Clínica Aplicada en Neoplàsies Hematològiques, Hospital del Mar Research Institute (IMIM), 08003 Barcelona, Spain; mabella@psmar.cat; 5 Laboratori de Citologia Hematològica, Servei de Patologia, Hospital del Mar, 08003 Barcelona, Spain; 6 Servei d’Hematologia Clínica, Hospital del Mar, 08003 Barcelona, Spain; 7 Servei de Patologia, Hospital del Mar, 08003 Barcelona, Spain; 8 Laboratori de Citogenètica Molecular, Servei de Patologia, Hospital del Mar, 08003 Barcelona, Spain

**Keywords:** Waldenström Macroglobulinemia (WM), peripheral blood involvement, flow cytometry, MFC, *MYD88* L265P, dPCR

## Abstract

**Simple Summary:**

Waldenström Macroglobulinemia (WM) is a lymphoplasmacytic lymphoma with bone marrow involvement and IgM monoclonal gammopathy. Detection of *MYD88* L265P mutation (*MYD88*mut) has a crucial contribution to the diagnosis, since >90% of WM cases harbor this mutation. No studies have focused specifically on peripheral blood (PB) involvement in this entity. We evaluate the incidence of leukemic involvement in WM by multiparametric flow cytometry (MFC) and molecular techniques (IGH rearrangement and *MYD88*mut) at diagnosis. Overall, 80/100 (80%) of the patients presented PB involvement by any technique. The presence of PB involvement by MFC in 50% of the patients supports the role of PB immunophenotyping as an objective tool in the evaluation of patients presenting with IgM monoclonal gammopathy and would reinforce the value of this approach in clinical practice, avoiding morbidity especially in cases of indolent clinical behavior and in frail patients.

**Abstract:**

Waldenström Macroglobulinemia (WM) is a lymphoplasmacytic lymphoma with bone marrow (BM) involvement and IgM monoclonal gammopathy. To date, no studies have focused specifically on peripheral blood (PB) involvement. In this study, 100 patients diagnosed with WM according to the World Health Organization (WHO) criteria were included based on the demonstration of *MYD88*mut in BM and the availability of PB multiparametric flow cytometry (MFC) analysis. Leukemic involvement by MFC was detected in 50/100 patients. A low percentage of mature small lymphocytes in PB smears was observed in only 15 cases. *MYD88*mut by AS-qPCR was detected in PB in 65/100 cases. In cases with leukemic expression by MFC, *MYD88*mut was detected in all cases, and IGH was rearranged in 44/49 cases. In 21/50 patients without PB involvement by MFC, molecular data were consistent with circulating disease (*MYD88*mut by AS-qPCR 3/50, IGH rearranged 6/50, both 12/50). Therefore, PB involvement by standard techniques was detected in 71/100 patients. *MYD88*mut was detected in PB by dPCR in 9/29 triple negative cases. Overall, 80% of the patients presented PB involvement by any technique. Our findings support the role of PB MFC in the evaluation of patients with IgM monoclonal gammopathy and provide reliable information on correlation with molecular features. The development of a feasible MFC assay may stand as an objective tool in the classification of mature B cell neoplasms presenting with IgM monoclonal gammopathy.

## 1. Introduction

Waldenström Macroglobulinemia (WM) is a lymphoplasmacytic lymphoma (LPL) with bone marrow (BM) involvement by small lymphocytes admixed with variable numbers of plasmacytoid lymphocytes and plasma cells accompanied by IgM monoclonal gammopathy of any concentration [1,2,3]. According to the Mayo Clinic criteria, patients diagnosed with WM with a serum IgM ≥ 30 g/L and/or ≥10% BM lymphoplasmacytic infiltration who do not require immediate treatment are classified as smoldering WM (SWM), and patients with serum IgM < 30 g/L and <10% BM infiltration with no symptoms or signs of end organ damage are classified as IgM monoclonal gammopathy of unknown significance (IgM MGUS) [4]. However, some groups reserve the diagnosis of IgM MGUS for patients without immunophenotypic evidence of WM clonal B cell population [5], as was stated in the Second International Workshop on Waldenström Macroglobulinemia [6], whereas the diagnosis of asymptomatic WM (AWM) could be established in a patient without symptoms regardless of the percentage of BM infiltration and the amount of IgM.

BM infiltration by WM has been classically assessed in BM trephine samples taking into account a combination of histologic characteristics, usually paratrabecular and/or interstitial infiltration by lymphoplasmacytoid cells and plasma cells with Dutcher bodies, as well as an increased number of mast cells [7,8]. Different studies have shown a good correlation between the presence of B lymphocyte infiltration assessed by immunohistochemistry on BM trephine or by multiparametric flow cytometry (MFC) in the BM aspirate [9]. The neoplastic cells of LPL/WM express CD19, CD20, IgM, and monotypic surface immunoglobulin light chain [10,11,12]. Paiva and colleagues described a characteristic WM phenotype CD22^+dim^/CD25^+^/CD27^+^/sIgM^+^ that differed from other mature B cell neoplasms by the absence of expression of CD5, CD10, CD11c, or CD103 [13]. However, it has been reported that CD5 expression can be observed in WM in approximately 5–10% of cases [14,15]. Nevertheless, the immunophenotype of WM clonal B lymphocytes easily overlaps with other B cell lymphoproliferative disorders, especially splenic marginal zone lymphoma (SMZL), which can present with monoclonal IgM, lymphoplasmacytic differentiation, and a non-specific B cell immunophenotype [16,17]. In these cases, the integration of morphologic, immunothenotypic, cytogenetic, molecular, and clinical data is mandatory.

From the molecular standpoint, *MYD88* L265P mutation (*MYD88*mut) was initially described as a highly specific molecular marker or LPL/WM, since >90% of cases harbor this mutation [18,19]. However, subsequent studies have pointed out that the presence of the mutation is not exclusive to LPL/WM, and there is a proportion of other small B cell lymphomas with lymphoplasmacytic differentiation in which *MYD88*mut can be present, mainly including MZL cases (4–21%) [20,21,22]. Even so, *MYD88*mut is a useful diagnostic tool that contributes to the diagnosis of LPL/WM.

Peripheral blood (PB) involvement in WM has been widely accepted as a frequent feature [1], but, to our knowledge, no studies to date have focused on this issue. In this context, we investigated the incidence of PB involvement in a large series of patients with WM by MFC analysis and correlated this feature with morphologic and molecular data in PB (immunoglobulin heavy chain (IGH) rearrangement by PCR, *MYD88* L265P status by allele-specific quantitative PCR (AS-qPCR) and by digital PCR (dPCR)).

## 2. Materials and Methods

### 2.1. Patients and Samples

One hundred patients diagnosed with WM from 2006 to 2021 in a single institution (Hospital del Mar, Barcelona, Spain) were included in this study based on the availability of PB analysis by MFC. Diagnosis of WM was established according to the World Health Organization (WHO) criteria and was carried out by cytologic, immunophenotypic, and genomic studies in BM. Patients were selected for this study only if small B cell lymphocytes, lymphoplasmacytoid cells, and plasma cells were observed in a BM aspirate and/or trephine biopsy, an abnormal B cell population was demonstrated by MFC, and *MYD88*mut was detected in BM. Of note, in all the patients included in this series, the presence of *MYD88*mut was demonstrated in BM in order to avoid the inclusion of other B lymphoproliferative disorders with plasmacytic differentiation and IgM monoclonal peak associated. In our institution, BM trephine biopsy was performed only in patients with symptomatic WM requiring therapy. Asymptomatic patients with less than 10% BM involvement in the BM aspirate and the presence of immunophenotypical findings of lymphoplasmacytic lymphoma were considered AWM. Informed consent was obtained from all patients in accordance with the local ethics committee guidelines.

The following initial data were recorded and evaluated for analysis in each patient: (1) clinical data (age, sex, performance status according to the Eastern Cooperative Oncology Group [ECOG] score, and symptoms present at diagnosis); (2) hematological and biochemical parameters (leukocyte and lymphocyte counts, hemoglobin level, platelet count, serum lactate dehydrogenase [LDH] and β2-microglobulin levels, albumin, serum monoclonal component, levels of IgM, IgG and IgA, and urine monoclonal component); (3) PB data (presence of atypical lymphocytes, immunophenotypic study of B lymphocytes by MFC, IGH rearrangement by PCR, *MYD88* L265P mutation status by AS-qPCR, and dPCR in selected cases); (4) BM data (presence of lymphocytes and plasma cells in the BM aspirate, MFC analysis of B lymphocytes and plasma cells, IGH rearrangement by PCR, *MYD88* L265P status by AS-qPCR, cytogenetics, and histological data in cases with BM trephine availability); (5) tumor extension data (lymph node and extranodal involvement, splenomegaly, and hepatomegaly); and (6) treatment requirement at presentation.

### 2.2. Morphologic, Immunophenotypic, and Molecular Studies

Smears of PB and BM were stained with May-Grünwald-Giemsa, whereas BM biopsy sections were stained with hematoxylin and eosin. The percentage and morphologic characteristics of lymphocytes and plasma cells were determined after counting a minimum of 200 or 500 cells per case in PB and BM aspirates, respectively. The pattern and extent of BM infiltration were also evaluated in trephine biopsies if available.

The presence of WM cells in PB was assessed by MFC. A minimum of 100,000 cells were acquired in a FACSCanto II flow cytometer (Becton-Dickinson, San Jose, CA, USA) and were analyzed with the Infinicyt software 2.0 (Cytognos, Salamanca, Spain). The 4- or 8-color MFC panel used in our laboratory routine as a first diagnostic approach in suspected B-cell lymphoproliferative disorders included the following monoclonal antibodies: CD5, CD10, CD11c, CD19, CD20, CD22, CD23, CD25, CD27, CD38, CD43, CD49d, CD79b, CD103, CD123, CD200, BCL2, FMC7, kappa, and lambda surface light chains. The gating strategy was based on the characteristic WM immunophenotypic features described by San Miguel et al. and Paiva et al. [12,13]. The evidence of a CD19^+dim^/CD22+^dim^/CD25^+^ B cell population with light chain restriction was considered diagnostic of leukemic expression. Moreover, the presence of a B cell population with a non-specific phenotype (different to that observed in chronic lymphocytic leukemia (CLL), mantle cell lymphoma, follicular lymphoma, and hairy cell leukemia) displaying light chain restriction was also considered indicative of PB involvement.

The study of the *MYD88* L265P mutation was performed by AS-qPCR [23,24] (7500Fast, Applied Biosystems, Woburn, MA, USA) and dPCR (QuantStudio 3D Digital PCR System, Applied Biosystems, Woburn, MA, USA). For IGH rearrangement analysis, PCR amplification of the FRI, FRII, and FRIII regions was performed [25], followed by PCR product analysis by capillary electrophoresis (3500 DX Series Genetic Analyzer, Applied Biosystems, Woburn, MA, USA).

### 2.3. Statistical Analysis

Categorical variables were described by frequencies and percentages, and continuous variables by medians and ranges. The Shapiro–Wilk Test was used to verify the normality of the sample. For categorical data, comparisons of proportions were evaluated by Chi-square test. For continuous variables, comparisons of medians, ranks, and means were assessed by parametric tests (*t*-Student) according to the distribution of the studied variables. *p*-values ≤ 0.05 were considered statistically significant. Cohen’s kappa coefficient (k) was used to compare the agreement between PB involvement between MFC, *MYD88* L265P mutation by AS-qPCR, and IGH rearrangement. Kendall’s τ coefficient was used to compare the agreement between PB involvement by MFC and the percentage of total CD19^+^ lymphocytes in PB, percentage of total CD19^+^ lymphocytes in BM, and percentage of clonal CD19^+^ lymphocytes in BM. Statistical analysis was performed by using SPSS 24.0 software (SPSS Inc., Chicago, IL, USA).

## 3. Results

### 3.1. Patients Characteristics

Table 1 shows the main characteristics of the whole series (N = 100 patients). The reason for referral was the detection of a monoclonal gammopathy in a routine PB test in asymptomatic patients in 83/100 (83%) cases. The remaining patients presented with symptoms: constitutional syndrome (5/17), anemia-related symptoms (3/17), polyneuropathy (3/17), hyperviscosity-related symptoms (3/17), or lymphadenopathy (1/17). The median leukocyte count was 7.08 × 10^9^/L, the median lymphocyte count was 2.00 × 10^9^/L, and the median percentage of B lymphocytes from the total lymphocyte count was 6%. Only 6/100 patients presented with a lymphocyte count ≥ 4 × 10^9^/L, whereas, in 6/100 patients, lymphopenia < 1.00 × 10^9^/L was observed. A total of 9 out of 100 patients presented with Hb ≤ 110 g/dL and 5/100 with platelets ≤ 150 × 10^9^/L. The median serum monoclonal component was 12.72 g/L (1.89–72.80 g/L).

### 3.2. Phenotypic Characteristics of Circulating WM Cells

**Figure 1:**
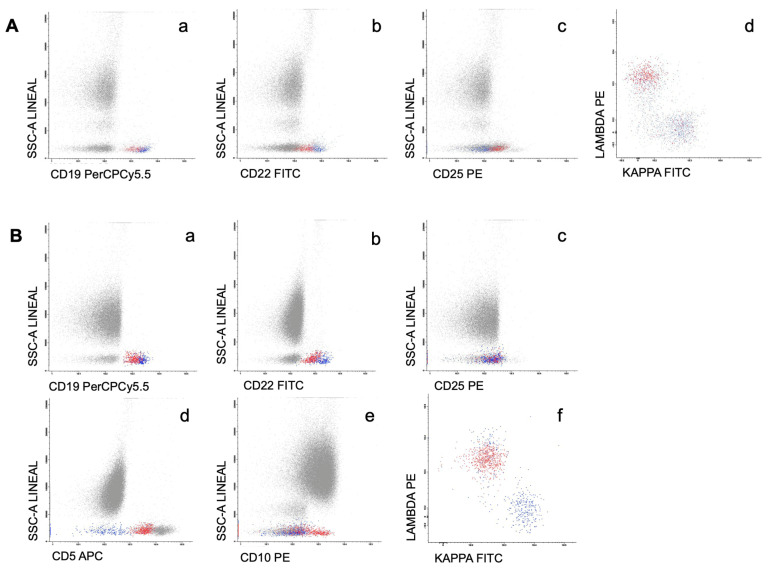
(**A**) Flow cytometry histograms of the PB in a patient with the phenotype usually described in WM. The figure shows the immunophenotypic profile of normal and abnormal B lymphocytes (colored in blue and red, respectively) regarding the expression of CD19 (**a**), CD22 (**b**), CD25 (**c**), and surface light chains (**d**). (**B**) Flow cytometry histograms of the PB in the CD19^+dim^/CD22^+dim^/CD25^−^ WM with co-expression of CD5 and CD10. The figure shows the immunophenotypic profile of normal and abnormal B lymphocytes (colored in blue and red, respectively) regarding the expression of CD19 (**a**), CD22 (**b**), CD25 (**c**), CD5 (**d**), CD10 (**e**), and surface light chains (**f**).

Leukemic involvement by MFC was detected in 50/100 patients (50%). The phenotype of abnormal B lymphocytes was the usually described in WM (CD22^+dim^/CD25^+^) in 20/50 cases (40%) (Figure 1A). In 15/50 patients (30%), a CD22^+dim^/CD25^−^ monotypic B cell population was observed. In 2/50 cases with CD22^+dim^, CD25 data were not available. In the remaining patients, a CD22^+^/CD25^+^ phenotype was observed in 7/50 cases, followed by a non-specific phenotype (CD22^+^/CD25^−^) in 6/50. CD5 expression was observed in 12/50 cases with PB involvement. CD10 was positive in one case, also co-expressing CD5 (Figure 1B). Of note, CD11c expression was detected in only one patient (CD22^+^/CD25^+^/CD11c^+^ phenotype). In all cases, monotypic surface light chain expression was required to consider PB expression.

**Figure 2:**
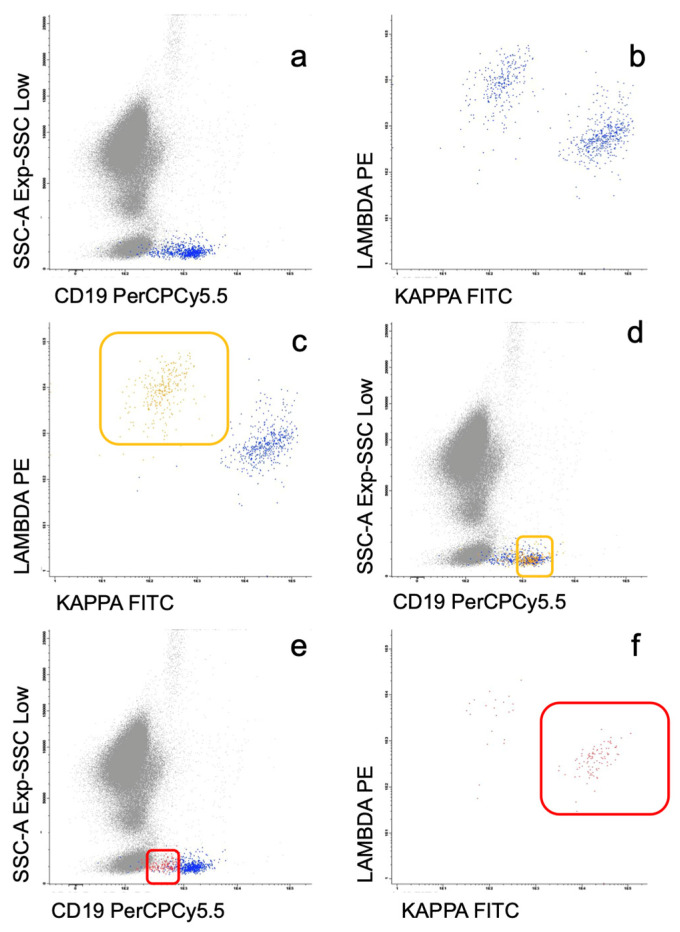
Flow cytometry histograms of the PB in a patient with monoclonal IgM kappa peak in which a small abnormal B cell population with normal kappa:lambda ratio (61% vs. 39%) was detected (**a**,**b**). When analyzing the population of B lymphocytes with uninvolved (non-clonal) light chain expression (CD19^+^lambda^+^, yellow frame), the intensity of CD19 expression was found to be higher than that observed in the remaining B lymphocytes (**c**,**d**). Only when the dimmer CD19^+^ B lymphocytes were analyzed (red frame), the kappa:lambda ratio was altered, displaying the presence of monotypic kappa^+^CD19^+^ neoplastic cells (82%) (**e**,**f**).

In 15 patients, the abnormal B cell population in PB was too small to be adequately discriminated from residual normal B cells, and a different approach during the analysis was required. In these cases, the neoplastic population was only detected in the kappa/lambda/CD19 tube, even when the kappa:lambda ratio was polytypic and apparently normal (75:25) for the whole CD19 population. Therefore, when analyzing the population of B lymphocytes with uninvolved (non-clonal) light chain expression (i.e, CD19^+^lambda^+^ cells in a patient with a monoclonal IgM kappa^+^ peak), the intensity of CD19 expression in these normal B lymphocytes was found to be higher than that observed in neoplastic B cells. In other words, only when the dimmer CD19^+^ B lymphocytes were analyzed, the kappa:lambda ratio was altered, displaying the presence of monotypic kappa^+^ or lambda^+^CD19^+^ cells consistent with an underrepresented monoclonal B cell population otherwise indistinguishable from the normal B lymphocytes (Figure 2).

In patients with PB involvement, the median of B cells in PB regarding the total lymphocytes was 7.5% (0.6–47%), with only nine patients presenting with ≥20% B lymphocytes. The median percentage of clonal B cells with respect to the total number of B cells was 60% (8–99%). However, in 15/50 patients, the clonal B lymphocytes could not be quantified because of their low representation, requiring the gating strategy described above (Figure 2). In 7/50 patients (14%), an additional population of abnormal B cells was detected, with 5/7 cases being phenotypically consistent with CLL.

### 3.3. Morphologic Features in WM with PB Involvement

**Figure 3:**
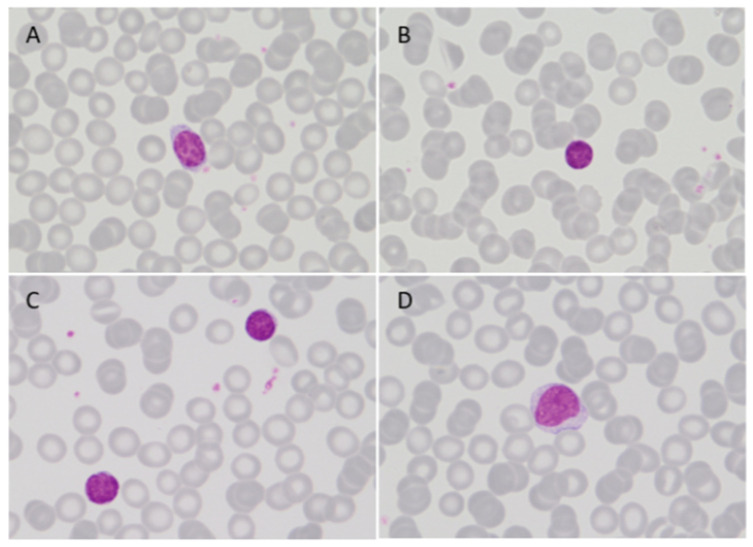
PB smear (MGG) in leukemic WM, 100×. Small lymphocytes with round nuclei, clumped chromatin, and scanty cytoplasm (**A**–**C**) or lymphocytes with moderately large, slightly basophilic cytoplasm and inconspicuous nucleolus (**D**) were observed.

In our daily practice, morphological examination of a PB smear is performed whenever an immunophenotypic study is requested. No circulating atypical lymphocytes were identified in the majority of patients with MW. In only 15 patients, a low percentage of small lymphocytes with clumped chromatin and scanty cytoplasm or lymphocytes with moderately large and basophilic cytoplasm were observed in PB (Figure 3). As expected, all of the patients with circulating atypical lymphocytes presented PB involvement by MFC. In order to better assess the morphologic appearance of WM circulating cells, leukemic patients with lymphocytes ≥ 4 × 10^9^/L (N = 6) and cases with high burden PB involvement by MFC (>30% B cells and >80% clonal CD19^+^/total CD19^+^ in PB, N = 5) were specifically reviewed. After a comprehensive revision, no differences were observed regarding the morphologic characteristics of the atypical lymphocytes described in the initial PB evaluation in these patients. Remarkably, the patient with the most evident leukemic expression had 13% of mature lymphocytes very similar to those observed in CLL. Of note, in any case, plasmacytoid lymphocytes, plasma cells, or villous lymphocytes were identified.

### 3.4. Molecular Data in Patients with and without Leukemic Expression by MFC

Overall, *MYD88*mut by AS-qPCR was detected in PB in 65/100 cases (65%). In cases with leukemic expression by MFC, *MYD88*mut by AS-qPCR was detected in PB in 100% of the cases, and IGH was rearranged in 44/49 cases (90%) (not available in one patient). In 23/50 patients (46%) without PB involvement by MFC, molecular data were consistent with circulating disease (only *MYD88*mut by AS-qPCR 3/50, only IGH rearranged 8/50, both 12/50). Nevertheless, in two patients in which PB IGH rearrangement was the unique feature suggestive of PB involvement by WM, the peak of the rearranged IGH was different from that observed in BM, indicating the presence of a circulating mature B cell neoplasm other than WM. Taking into account all these findings, PB involvement by standard techniques was detected in 71/100 patients. The Cohen’s kappa statistic (k) showed a good correlation between MFC and *MYD88*mut by AS-qPCR (k = 0.700) and a moderate correlation between MFC and IGH gene rearrangement (k = 0.476). The results are summarized in the Table 2.

A group of 29 cases were considered triple negative: absence of abnormal B lymphocytes in PB by MFC, *MYD88*wt by AS-qPCR, and non-rearranged IGH (or rearranged IGH different from the observed in BM in the context of additional abnormal B cell populations in PB). A similar distribution of triple negative cases was observed between symptomatic WM cases (5/17; 23.5%) and AWM patients (24/83; 29%). To ensure absence of *MYD88* L265P mutation in this group with the most sensitive technique, dPCR was performed [26,27]. *MYD88*mut was detected by dPCR in 9/29 triple negative cases (31%).

**Figure 4:**
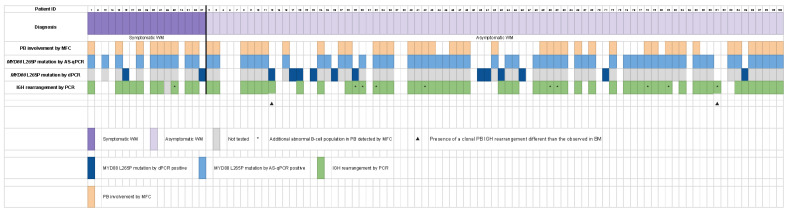
The heatmap shows the data from the WM patients of the series (N = 100). Each column represents a patient. On the top left of the heatmap, the patients with symptomatic WM are shown in dark violet, and, on the top right, the patients with asymptomatic WM are depicted in light violet. Each row represents (from top to bottom): PB involvement by MFC (positive cases are represented as orange cells and negative cases as white cells); *MYD88* L265P mutation by AS-qPCR (positive cases are depicted in blue and negative cases in white); *MYD88* L265P mutation by dPCR (positive cases are represented as dark blue cells and negative cases as white cells), and IGH rearrangement by PCR (positive cases are depicted in green and negative cases in white). The presence of additional abnormal circulating B cell populations is represented by a black star. The presence of a clonal PB IGH rearrangement different than that observed in BM is depicted with a black triangle. Grey cells indicate not tested.

**Figure 5:**
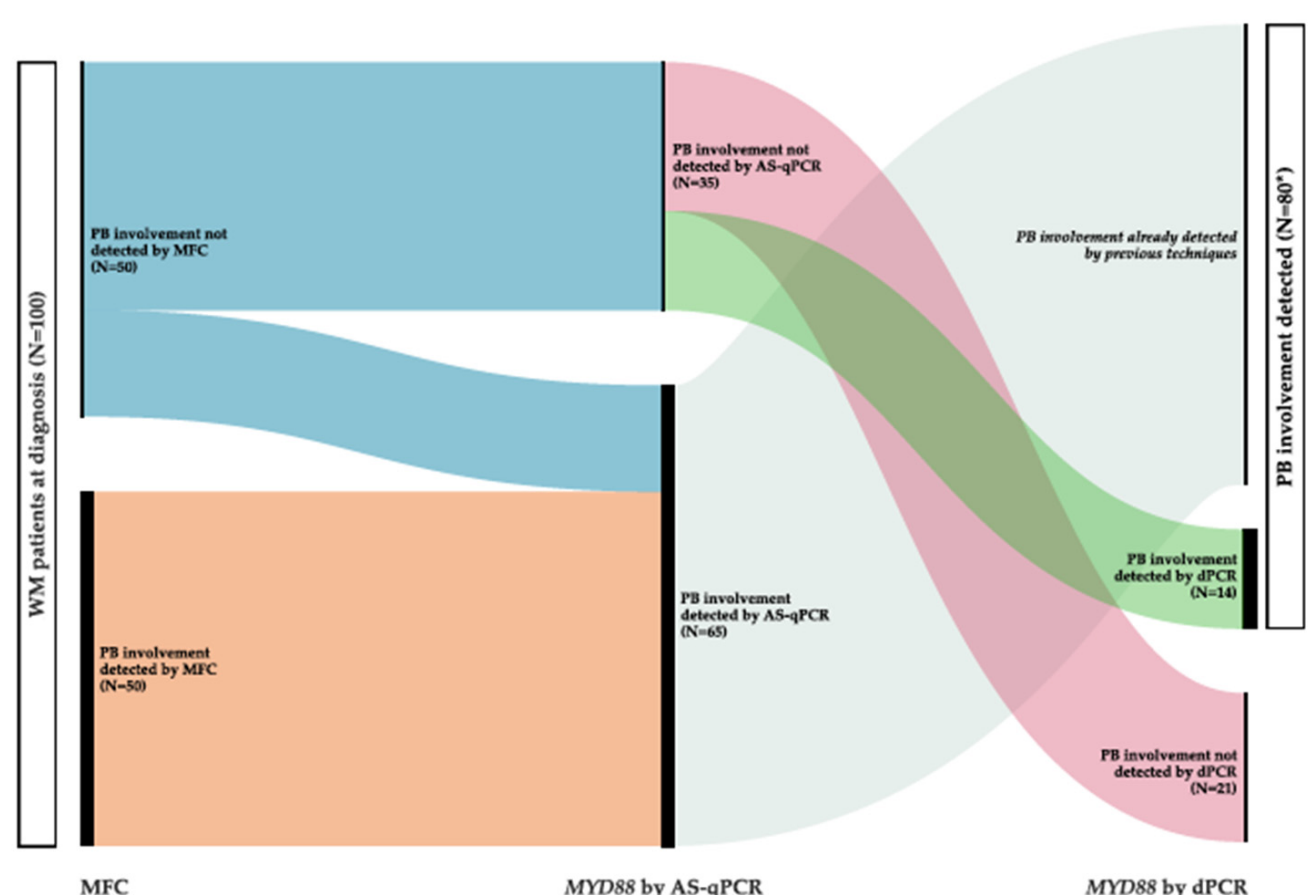
Alluvial diagram showing the cases with PB involvement by MFC (n = 50), all of them with *MYD88*mut by AS-qPCR (100%). In the cases with no evidence of leukemic involvement by MFC (n = 50): *MYD88*mut by AS-qPCR in 15/50 and *MYD88wt* by AS-qPCR in 35/50 patients. *MYD88* L265P mutation by dPCR was performed in the latter group; in 14/35 patients, *MYD88*mut was detected by dPCR and not detected in 21/35 patients. * In one patient, PB involvement was detected by IGH rearrangement only.

Globally, 80/100 (80%) of the patients presented PB involvement by any technique (MFC and molecular studies, including dPCR) while 20/100 (20%) were negative by all four techniques. The whole immunophenotypic and molecular PB data from the cohort are outlined in Figure 4. Figure 5 summarizes the correlation between MFC and the *MYD88* L265P mutational status by AS-qPCR and dPCR.

### 3.5. Correlation between Leukemic Expression, Clinical and Analytical Data, and Bone Marrow Features

No differences were observed between leukemic and non-leukemic patients by MFC regarding clinical characteristics such as age (*p* = 0.303), sex (*p* = 0.685), ECOG > 1 (*p* = 1.000), adenopathy (*p* = 1.000), splenomegaly (*p* = 1.000), and treatment requirement at diagnosis (*p* = 1.000). In addition, no differences in analytical data such as leukocyte count (*p* = 0.135), lymphocyte count (*p* = 0.110), hemoglobin level (*p* = 0.701), platelet count (*p* = 0.849), LDH level (*p* = 0.362), and β2-microglobulin level (*p* = 0.100) were detected between both groups. PB involvement by MFC was not related to the levels of serum IgM monoclonal protein (*p* = 0.397). No differences were observed between the presence of leukemic expression and positive urine immunofixation (*p* = 0.550).

PB involvement by MFC was related to the percentage of total CD19^+^ lymphocytes in PB (*p* = 0.001) as well as in BM (*p* = 0.006) and to the percentage of clonal CD19^+^ lymphocytes in BM (*p* = 0.046). Kendall’s τ coefficient was used to assess the correlation between PB involvement by MFC and the percentage of total CD19^+^ lymphocytes in PB (τ = 0.289; *p* = 0.001), the percentage of total CD19^+^ lymphocytes in BM (τ = 0.223; *p* = 0.007), and the percentage of clonal CD19^+^ lymphocytes in BM (τ = 0.185; *p* = 0.041). Moreover, leukemic involvement by MFC was most frequently observed in patients displaying ≥20% lymphocytes in BM by morphology (34/49, 69.3% vs. 15/49, 30.6% in patients with <20% lymphocytes, *p* = 0.001). On the contrary, the percentage of plasma cells in BM by morphology and MFC was not related to the presence of leukemic involvement (*p* = 0.811). Of note, in two patients, the quality of the BM aspirate did not allow morphologic assessment.

When specifically analyzing patients with no evidence of disease in PB by any of the four techniques used (MFC, IGH rearrangement, L265P *MYD88*mut by AS-qPCR, or dPCR), no differences were observed between leukemic patients and non-leukemic patients regarding age (*p* = 0.446), sex (*p* = 0.268), ECOG > 1 (*p* = 0.384), lymphadenopathy (*p* = 0.585), splenomegaly (*p* = 0.620), and treatment requirement at diagnosis (*p* = 0.769). Patients without PB involvement by any technique had a lower a leukocyte count (*p* = 0.021), a lower CD19^+^ lymphocyte count in PB (*p* = 0.017), and a lower CD19^+^ lymphocyte count in BM (*p* = 0.032) compared to the leukemic group. No differences were observed regarding lymphocyte count (*p* = 0.680), hemoglobin level (*p* = 0.459), platelet count (*p* = 0.153), LDH level (*p* = 0.505), and β2-microglobulin level (*p* = 0.109). No differences were observed regarding the amount of serum monoclonal component (*p* = 0.883), the positivity of urine immunofixation (*p* = 0.573), the percentage of clonal CD19^+^ lymphocytes in BM (*p* = 0.540), and the percentage of plasma cells by morphology (*p* = 0.507) or by MFC (*p* = 0.624).

Finally, treatment requirement at diagnosis was not related to PB involvement by MFC (*p* = 1.000) or to any other technique (*p* = 1.000), neither was it related to the amount of clonal CD19^+^ lymphocytes in PB (*p* = 0.842), the amount of CD19^+^ lymphocytes in BM (*p* = 0.482), or the levels of serum monoclonal component (*p* = 0.056). Patients with a higher percentage of clonal CD19^+^ lymphocytes in BM required treatment at diagnosis more frequently (*p* = 0.016).

## 4. Discussion

Although PB involvement has been usually considered a frequent feature of WM, to our knowledge, there are no studies that directly address this issue. According to the Second International Workshop on Waldenström Macroglobulinemia [6] and, as recently stated by Bustoros et al. [5], immunophenotypic evidence of WM B cell population by MFC was detected in BM in all our patients. In addition, only cases with *MYD88* L265P mutation in BM were included in order to rule out other B-cell disorders with plasmacytic differentiation and an associated IgM monoclonal peak. Therefore, all patients included in the present series had been homogeneously studied and diagnosed with WM after carefully excluding any alternative diagnosis. In our study, PB involvement by MFC was present in half of the patients diagnosed with MW. It is important to note that only 4/50 cases presented with a lymphocyte count ≥4 × 10^9^/L and that, in 42/50 patients, the percentage of B cells was <20% of all lymphocytes. From a morphological point of view, and unlike what occurs in the majority of mature B-cell neoplasms with PB involvement, circulating WM cells have a nonspecific appearance. As a whole, neither the presence of lymphocytosis nor the lymphocyte morphology in PB were useful to suspect the presence of leukemic disease.

The most frequent abnormal immunophenotype of the WM population in PB was the CD22^+dim^/CD25^+^ previously described by Paiva et al. [13], but more than half of the cases showed a different pattern. In addition, the detection of leukemic involvement by MFC can be even more challenging, as 30% of our cases presented with a very small clonal population that could not be quantified. In these cases, a different MFC approach was necessary, separately analyzing the CD19^+dim^ region and assessing whether the initially polyclonal population showed kappa or lambda restriction (Figure 2). Since leukemic MZL (mainly SMZL) is the mature B-cell neoplasm that most frequently displays a nonspecific phenotype, and up to 20% of such cases may present *MYD88* L265P mutation, differential diagnosis with SMZL was made with special thoroughness. In this regard, SMZL usually presents with lymphocyte counts ≥4 × 10^9^/L, atypical lymphocytes are easily recognized in the morphological study of PB, the median percentage of B lymphocytes from the total lymphocyte count is usually >20%, and the percentage of clonal CD19/total CD19 in PB is almost always higher than in WM. On top of that, the absence of villous lymphocytes in PB and the presence of a CD22^+dim^/CD25^+^/CD11c^−^ B cell population by MFC [16,28,29] supports the diagnosis of WM. In our experience, CD11c expression is observed in about 50% of leukemic SMZL. On the contrary, although CD11c was previously reported as commonly expressed in LPL/WM [15], CD11c positivity was only detected in one patient of our series, in line with more recent works [13]. In this particular case, the presence of a CD22^+^/CD25^+^/CD11c^+^ phenotype together with L265P *MYD88* mutation did not allow us to exclude, with complete certainty, the diagnosis of *MYD88*mut SMZL. Moreover, the absence of characteristic SMZL cytogenetics [30,31] and the integration of clinical data is essential in these especially difficult cases.

Regarding molecular data, the application of standard molecular techniques (*MYD88* L265P mutation by AS-qPCR and IGH rearrangement) revealed PB involvement in 23/50 WM cases (46%) in cases where leukemic involvement was not detected by MFC. The assessment of *MYD88* L265P mutation by dPCR in the triple negative cases according to standard techniques enabled the detection of nine additional patients with PB involvement. Thus, in cases without PB involvement by MFC and L265P *MYD88*wt by AS-qPCR, the dPCR showed higher sensitivity, as has been recently published [26]. Overall, only 20/100 patients presented a non-leukemic WM.

The diagnosis of WM requires the presence of IgM monoclonal gammopathy of any concentration and a BM study to demonstrate lymphoplasmacytic infiltration. In some mature B cell neoplasms with leukemic expression and indolent behavior, PB analysis is enough to establish the diagnosis, as is in the case of CLL [32]. Our study raises the possibility of establishing the diagnosis of WM based on the information provided by MFC and *MYD88* L265P status in PB in some patients, especially in those who are frail due to age and/or comorbidities, and in those who are asymptomatic and do not require immediate treatment. In this regard, Xu et al. [33] showed the feasibility and the potential for PB *MYD88* L265P detection in the diagnosis and management of WM patients, avoiding morbidity and saving time in comparison with BM-based diagnosis. The addition of PB immunophenotypic study in patients with IgM monoclonal gammopathy, especially in the 8–12 colors MFC era, also allows us to carry out an extensive differential diagnosis between WM and other leukemic mature B cell neoplasms.

**Figure 6:**
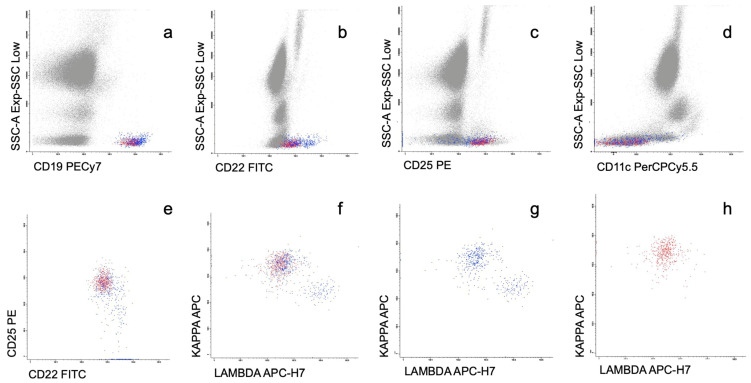
Representative flow cytometry histograms of WM patients using the eight-color tube implemented in our laboratory daily routine for detection of abnormal circulating B cells in cases with IgM monoclonal peak. The figure shows the immunophenotypic profile of normal and abnormal B cells (colored in blue and red, respectively) regarding the expression of CD19 (**a**), CD22 (**b**), CD25 (**c**), CD11c (**d**), and surface light chains (**f**–**h**). The presence of a CD22^+^dim/CD25^+^ population is better identified in a CD22/CD25 biparametric histogram (**e**). Surface light chain expression in the whole B cell population, in normal B lymphocytes, and in circulating WM cells is depicted in f (85% kappa^+^, 15% lambda^+^), g (70% kappa^+^, 30% lambda^+^) and h plots (100% kappa^+^), respectively.

In light of our results, we developed a single eight-color MFC tube to better assess PB involvement (Table 3, Figure 6). Additionally, we increased the number of acquired events to 200,000 for obtaining reliable detection rates, since the abnormal population was sometimes present in a very low percentage. From June 2021 to January 2023, 18 PB MFC studies from patients with an IgM monoclonal gammopathy displaying *MYD88* L265P mutation in PB were identified. Among them, 13 cases showed evidence of an abnormal B cell population with monotypic surface light chain expression. With this new approach, based on the combination of different markers that allow for better discrimination of the population of interest and the acquisition of a higher number of events, the percentage of detection increased to 72%. Thus, the detection of PB involvement by MFC depends on the sensitivity of the immunophenotypic approach.

One of the limitations of our study is the low incidence of patients with symptomatic WM and, therefore, in the need for starting treatment at diagnosis. Therefore, we do not have sufficient data to establish whether the presence of leukemic expression at diagnosis is related to the clinical course of the disease. Possibly, the incorporation of PB studies based on high sensitivity MFC and molecular techniques in a larger series of cases will help clarify if leukemic expression is an intrinsic feature in WM with no prognostic implications or if there are clinical and prognostic differences between both groups depending on the presence of PB involvement.

## 5. Conclusions

In conclusion, in the current series of patients with WM, leukemic expression detectable by MFC and molecular techniques at the time of diagnosis was a common feature even in patients with a normal lymphocyte count. Our findings support the value of performing PB MFC immunophenotyping in the assessment of patients with IgM monoclonal gammopathy and provide reliable information about the correlation with molecular features. Since the immunophenotype of circulating WM cells is not as nonspecific as it might first appear, the development of a single and feasible eight-color MFC tube may emerge as a reproducible and objective tool in the discrimination of mature B cell neoplasms that present with IgM monoclonal gammopathy. If this test could be applied along with molecular screening methods in prospective studies of a larger series of patients, it would reinforce the value of our approach as a new diagnostic tool of WM in clinical practice.

## Figures and Tables

**Table 1:** Baseline characteristics of the patients diagnosed with Waldenström Macroglobulinemia (N = 100).

	All Patients N = 100	Patients with PB Involvement by MFC N = 50 (50%)	Patients without PB Involvement by MFC N = 50 (50%)	Significance (*p*)
Median age, years (range, IQR)	73 (36–97) (IQR: 12.75)	73 (54–89) (IQR: 14)	73.5 (36–97) (IQR: 12.5)	*p* = 0.303 ^2^
Sex female/male	69.5%	78.6%	61.3%	*p* = 0.685 ^1^
ECOG > 1	3%	2%	4%	*p* = 1.000 ^1^
Lymphadenopathy (by physical examination and/or CT scan)	8%	8%	8%	*p* = 1.000 ^1^
Splenomegaly (by physical examination and/or CT scan)	1%	0%	2%	*p* = 1.000 ^1^
Leukocytes (×10^9^/L), median (range, IQR)	7.08 (3.37–17.58) (IQR: 3.10)	7.29 (3.97–17.58) (IQR: 3.08)	6.93 (3.37–17.53) (IQR: 3.63)	*p* = 0.135 ^2^
Lymphocytes (×10^9^/L), median (range, IQR)	2.00 (0.61–8.81) (IQR: 0.97)	2.3 (0.77–7.28) (IQR: 1.09)	1.87 (0.61–8.81) (IQR: 0.89)	*p* = 0.110 ^2^
Lymphocytes ≥ 4 × 10^9^/L	6%	8%	4%	*p* = 0.678 ^1^
Hemoglobin (g/L), median (range, IQR)	133 (82–178) (IQR: 23.75)	135 (82–167) (IQR: 20.00)	131 (88–178) (IQR: 26.25)	*p* = 0.701 ^2^
Platelets (×10^9^/L), median (range, IQR)	247 (110–669) (IQR: 104.75)	254 (110–503) (IQR: 109.00)	243 (124–669) (IQR: 101.25)	*p* = 0.849 ^2^
Increased serum LDH levels	5%	2%	8%	*p* = 0.362 ^1^
Increased serum β_2_-microglobulin levels	40.8%	50%	32%	*p* = 0.100 ^1^
Serum monoclonal component (g/L), median (range, IQR)	12.72 (1.89–72.80) (IQR: 6.36)	12.36 (5.93–43.50) (IQR: 5.38)	13.63 (1.89–72.80) (IQR: 7.30)	*p* = 0.397 ^2^
Serum monoclonal component ≥ 15 g/L	33%	26%	40%	*p* = 0.202 ^1^
Serum monoclonal component ≥ 30 g/L	5%	6%	4%	*p* = 1.000 ^1^
Positive urine immunofixation	25%	30.3%	19.3%	*p* = 0.550 ^1^
PB involvement by MFC	50%	-	-	-
Median % CD19 from total lymphocytes in PB (range, IQR)	6.00 (0.6–47) (IQR: 6.75)	7.5 (0.6–47) (IQR: 11.10)	5.00 (0.7–32) (IQR: 4.70)	***p* = 0.001** ^2^
Median % clonal CD19 from total CD19 in PB (range, IQR)	-	60 (8–99) (IQR: 60)	-	-
CD5 expression in PB in leukemic cases	-	24%	-	-
Median % CD19 from total lymphocytes in BM (range, IQR)	34 (4–76) (IQR: 25)	35 (5–76) (IQR: 22)	23,50 (4–60) (IQR: 23)	***p* = 0.006** ^2^
Median % clonal CD19 from total CD19 in BM (range, IQR)	80 (0–100) (IQR: 45)	90 (6–100) (IQR: 34)	70 (0–100) (IQR: 52)	***p* = 0.046** ^2^
BM lymphocytes, % (range)	22 (8–91)	28 (8–91)	18 (10–47)	***p* = 0.008** ^2^
BM plasma cells, % (range)	4 (0–18)	3 (1–15)	4 (0–18)	*p* = 0.811 ^2^
Treatment at presentation	13%	14%	12%	*p* = 1.000 ^1^

Abbreviations: IQR, interquartile range; ECOG, Eastern Cooperative Oncology Group; CT scan, computed tomography scan; LDH, lactate dehydrogenase; PB, peripheral blood; MFC, multiparametric flow cytometry; BM, bone marrow. Evaluated by: ^1^ Chi square or ^2^ *t*-Student.

**Table 2:** Correlation between PB involvement by MFC, *MYD88* L265P mutation by AS-qPCR and IGH gene rearrangement.

		PB Involvement by MFC Detected 50/100 (50%)	PB Involvement by MFC Not Detected 50/100 (50%)
*MYD88* L265P mutation by AS-qPCR in PB	*MYD88*mut	50/50 (100%)	15/50 (30%)
	*MYD88*wt	0/50 (0%)	35/50 (70%)
IGH gene rearrangement in PB	IGH rearranged	44/49 * (90%)	20 **/50 (40%)
	IGH non rearranged	5/49 * (10%)	30/50 (60%)

Abbreviations: PB, peripheral blood; MFC, multiparametric flow cytometry; AS-qPCR, allele-specific quantitative PCR; IGH, immunoglobulin heavy chain. * Not available in one patient. ** In two patients in which PB IGH rearrangement was the unique feature suggestive of PB involvement by WM, the peak of the rearranged IGH was different from that observed in BM, indicating the presence of a circulating mature B cell neoplasm other than WM.

**Table 3:** Composition of the eight-color tube for detection of WM circulating cells.

FITC	PE	PerCPCy5.5	PECy7	APC	APC-H7	V-450	V-500
CD22	CD25	CD11c	CD19	Kappa	Lambda	CD20	CD45

Abbreviations: FITC, fluorescein isothiocyanate; PE, phycoerythrin; PerCPCy5.5, peridinin–chlorophyll–protein–cyanin5.5; Cy7, cyanin7; APC, allophycocyanin; H7, hilite7.

## Data Availability

Data available on request from the corresponding author.
